# A new family of the order Monstrilloida (Copepoda) from deep waters of the North Atlantic supported by morphological and genetic evidence

**DOI:** 10.7717/peerj.21176

**Published:** 2026-05-18

**Authors:** Eduardo Suárez-Morales, Alejandro Martínez, Pedro Martinez Arbizu, Sahar Khodami, Nancy F. Mercado-Salas

**Affiliations:** 1 Unidad Chetumal, El Colegio de la Frontera Sur, Chetumal, Quintana Roo, Mexico; 2 Molecular Ecology Group (MEG), Water Research Institute (IRSA), National Research Council of Italy (CNR), Verbania, Italy; 3 German Centre for Marine Biodiversity Research, Senckenberg am Meer, Wilhelmshaven, Lower Saxony, Germany; 4 Centre for Taxonomy and Morphology, Museum of Nature Hamburg, Zoology, Leibniz Institute for the Analysis of Biodiversity Change, Hamburg, Germany

**Keywords:** Phylogeny, Systematics, 28S rRNA, 18S rRNA, Benthopelagic, mtCOI, CLSM, Deep-sea

## Abstract

**Background:**

Monstrilloid copepods have endoparasitic naupliar and postnaupliar stages infecting different groups of marine invertebrates. As adults, they have been recorded as free-swimming, non-feeding planktonic organisms in a wide variety of near-surface coastal and transitional aquatic habitats. The phylogenetic relations of the Monstrilloida, obscured by the lack of antennae and mouthparts, have long been a matter of discussion among copepodologists.

**Methods:**

Epibenthic samples collected at 2,537 m depth from the Irminger Basin, North Atlantic, yielded an adult male monstrilloid copepod that is unassignable to the Monstrillidae, the only known family of the order Monstrilloida. Herein, we erect a new family of the Monstrilloida to accommodate this individual. We provide a comprehensive morphological description of this specimen, along with a phylogenetic analysis that incorporates three genetic markers (mtCOI, 28S rRNA, and 18S rRNA), which supports the designation of the new family.

**Results:**

The most striking character in the new family is the remarkably long, slender antennules that are directed posteriorly, thus diverging from the typical monstrillid pattern with rigid, anteriorly directed antennules. Together with the large furca, these characters are considered traits for their adult planktonic life. Another striking character is the presence of a pair of indistinctly segmented, likely vestigial, appendages flanking the oral protuberance, instead of the absence of oral appendages typical of monstrillids. Also, the presence of biramous male fifth legs has never been observed in other monstrilloids, in which male fifth legs are absent or reduced. The distinctive morphological characters exhibited by this new family, along with its deep-sea benthopelagic occurrence, could provide new elements for re-evaluating the phylogenetic position of the Monstrilloida, as well as insights into their biology and ecology.

## Introduction

Monstrilloid copepods are internal parasites during their larval stages capable of infecting different groups of marine invertebrates, including polychaetes, molluscs, and sponges (Suárez-Morales, 2011; Suárez-Morales, 2018; Jeon, Lee & Soh, 2018; Emelianenko et al., 2025). They are frequently recorded as non-feeding planktonic adults in a wide variety of near-surface coastal and transitional aquatic habitats (Suárez-Morales, 2018; Cruz Lopes da Rosa et al., 2021), but they were recently reported from epi-mesopelagic depths (Suárez-Morales & Mercado-Salas, 2023; Suárez-Morales & Velázquez-Ornelas, 2024). Their taxonomic and nomenclatural history is complex and remains largely unresolved (Grygier, 1995; Grygier & Ohtsuka, 2008; Grygier & Suárez-Morales, 2018; Suárez-Morales, 2011; Suárez-Morales, 2018; Suárez-Morales & Grygier, 2021), particularly in reference to old records, incomplete early species descriptions, and the difficulties to reliably matching males and females of the species (Grygier, 1993; Grygier, 1995; Suárez-Morales, 2010; Suárez-Morales, 2011; Suárez-Morales, 2018; Suárez-Morales & Grygier, 2021).

The order Monstrilloida Sars, 1901 is currently represented by a single family (Monstrillidae Dana, 1849–1852) containing eight valid genera: *Monstrilla* Dana, 1849; *Cymbasoma* Thompson, 1888; *Monstrillopsis* Sars, 1921; *Maemonstrilla* Grygier & Ohtsuka, 2008; *Australomonstrillopsis* Suárez-Morales & McKinnon, 2014; *Caromiobenella* Jeon, Lee & Soh, 2018, *Spinomonstrilla* Suárez-Morales, 2019, and *Sarsimonstrillus* Suárez-Morales & McKinnon, 2025; (Suárez-Morales, 2011; Suárez-Morales, 2019; Suárez-Morales & McKinnon, 2014; Suárez-Morales & McKinnon, 2025; Jeon et al., 2024; Walter & Boxshall, 2025). They share the diagnostic characters of the order Monstrilloida and family Monstrillidae including: (1) rigid, anteriorly directed antennules usually shorter than cephalothorax, (2) 5-segmented male antennules, (3) oral structures absent, represented only by an oral protuberance, (4) legs 1–4 with 3-segmented endopods and exopods, (5) male fifth leg reduced or absent (Huys & Boxshall, 1991; Boxshall & Halsey, 2014; Jeon et al., 2024).

An epibenthic sample collected in deep-sea waters of the North Atlantic Ocean during the IceAge Expedition (M 85/3) contained a single adult male specimen of a monstrilloid copepod that could not be accommodated in the Monstrillidae, the only existing family of the order Monstrilloida (Huys & Boxshall, 1991; Suárez-Morales, 2011; Suárez-Morales, 2018; Boxshall & Halsey, 2014; Jeon et al., 2024). This remarkable individual is here assigned to a new monstrilloid family, which is here described and morphologically distinguished from the Monstrillidae. Accompanying genetic data are herein analysed to provide additional evidence supporting our proposal of this new taxon.

## Material & Methods

### Sample collection

The specimen described in this study was collected during the cruise M 85/3 IceAge (2011) on board the FS *Meteor*, which was part of the project Icelandic marine Animals: Genetics and Ecology (IceAGE) (Brix et al., 2014). The sample containing the specimen examined in this work was obtained with an epibenthic sledge (EBS) operated in deep water of the Iceland basin. The EBS was equipped with two nets, including an upper supranet and a lower epinet, both with a filtering mesh size of 500 µm and a valve to retain the collected material (Brencke, 2005), thus ensuring that the specimen was collected at the stated depth (see also Suárez-Morales & Mercado-Salas, 2023). After collection, the sample was fixed in 96% Ethanol and kept at −20 °C until further processing.

### Morphological description

Separation and preliminary observations of the specimen under study were made under an Olympus SZ51 stereomicroscope, and further taxonomic examination was made with an Olympus BX51 with Nomarski Differential Interference Contrast (DIC). Prosome, urosome, and an antennule were also examined with a Leica TCS-SPE confocal laser scanning microscope (CLSM) at the Zoological Institute of the University of Hamburg. The specimen was processed following the CLSM methodology described by Mercado-Salas et al. (2018) (modified from Michels & Büntzow (2010)). After CLSM examination, the specimen was mounted in seven permanent slides. The slides were deposited in the Crustacea Collection (ZMH-K-066757) held at the Museum of Nature Hamburg, Zoology, Germany (Leibniz Institute for the Analysis of Biodiversity Change). The general morphological terminology follows Huys & Boxshall (1991). The nomenclature of the antennular setation pattern follows Grygier & Ohtsuka (1995). The term ‘furca’ follows Schminke (1976).

### Nomenclatural acts

The electronic version of this article in Portable Document Format (PDF) will represent a published work according to the International Commission on Zoological Nomenclature (ICZN), and hence the new names contained in the electronic version are effectively published under that Code from the electronic edition alone. This published work and the nomenclatural acts it contains have been registered in ZooBank, the online registration system for the ICZN. The ZooBank LSIDs (Life Science Identifiers) can be resolved and the associated information viewed through any standard web browser by appending the LSID to the prefix http://zoobank.org/. The LSID for this publication is: urn:lsid:zoobank.org:pub:4092F952-8C15-454D-9924-189ADF3B2A6D. The online version of this work is archived and available from the following digital repositories: PeerJ, PubMed Central SCIE and CLOCKSS.

### DNA extraction and amplification

Prior to morphological work, DNA was extracted using the EZNA^®^ Mollusc DNA Kit (Omega Bio-Tek, Norcross, GA, USA) following the manufacturer’s protocol. To avoid the specimen’s damage, lysis was performed overnight at 37 °C. After lysis, the cuticle was recovered by transferring the supernatant to a new tube to continue the extraction, whilst the original tube containing the cuticle was filled with 96% ethanol and used for morphological work. The gene 18S rRNA was amplified with the universal primers 18SE-F (Hillis & Dixon, 1991) and 18SL-R (Hamby & Zimmer, 1988); for sequencing, internal primers F1, CF2, CR1, and R2 (Laakmann et al., 2013) were also used. The 28S rRNA was amplified using 28S-F1a and 28S-R1a primer pairs (Ortman, 2008). Additionally, COI marker was amplified using the primer pairs Cop-COI-2189R (Bucklin et al., 2010) and jgLCO (Geller et al., 2013). Amplification of all markers (18S, 28S, and COI) was performed following Suárez-Morales & Mercado-Salas (2023) using AccuStart GelTrack PCR SuperMix (ThermoFisher Scientific, Waltham, MA, USA) in a 20 μL volume containing 10 μL AccuStart, seven μL H2O, 0.5 μL of each primer (10 pmol/μL), and two μL of DNA template. The Polymerase Chain Reaction (PCR) protocol for 18S and 28S genes was 94 °C for 3 min, 94 °C for 30 s, 47 °C for 1 min, and 72 °C for 1 min, for 35 cycles, followed by a final elongation at 72 °C for 1 min. For the COI marker, the PCR protocol was 94 °C for 3 min, then 94 °C for 30 s, 45 °C for 45 s, and 72 °C for 1 min, for 35 cycles, followed by a final elongation at 72 °C for 2 min. All PCR products were checked by electrophoresis on a 1% agarose/TAE (tris-acetate-EDTA) gel containing 1% GelRed, and successful PCR products were purified using ExoSap-IT PCR Product Cleanup (Affymetrix, Inc., Santa Clara, CA, USA). Sequencing was carried out by Macrogen (Amsterdam, the Netherlands). Geneious 9.1.7 (created by Biomatters, Auckland, NZ) was used to assemble, edit, check for indels, and translate sequences into amino acids to confirm the absence of stop codons. GenBank and BOLD accession numbers are given in Table 1.

**Table 1:** GenBank and BOLD accession numbers (AN) of *Thalassodoron bathyale* n. gen et n. sp.

**Collection number**	**DNA extraction ID**	**GenBank AN 18S rRNA**	**GenBank AN 28S rRNA**	**BOLD AN mtCOI**
ZMH-K-066757	IA318	PX412398	PX434333	IACOP001-25

### Molecular phylogenetic analyses

Following the morphological description, we investigated the phylogenetic position of the collected individual based on three molecular markers (18S rRNA, 28S rRNA, and mtCOI), using all relevant sequences available in GenBank. We downloaded all Monstrilloida sequences and associated metadata using the function *download.refs* from the NGSmeioR pipeline (Martínez, in preparation), obtaining 158 sequences. Of these, 157 corresponded to the targeted markers (28, 18S rRNA; 63, 28S rRNA; and 66, mtCOI). From those, 106 were identified to species level and 53 to genus level, covering a total of 13 different formally described species and five genera out of the eight described for the order (Walter & Boxshall, 2025). Outgroups included one calanoid, two mormonilloids, and four siphonostomatoids. A complete list of sequences, accession numbers, and metadata is provided in Supplemental Information 1.

Each marker was aligned separately, including sequences from our newly collected specimen. For 18S and 28S rRNA, we used the E-INS-i algorithm due to the presence of conserved domains interspersed with variable regions; for mtCOI, we allowed MAFFT to select the best strategy automatically (Strategy: auto). The mtCOI alignment was inspected for indels and translated into amino acids to detect stop codons. Ambiguous sequences were excluded from further analysis. Maximum Likelihood (ML) trees for each marker were inferred using IQ-TREE (http://iqtree.cibiv.univie.ac.at/), employing the GTR+G substitution model and Ultrafast bootstrap support.

Final phylogenetic analyses were based on a concatenated dataset. Initially, sequences from the same individual were concatenated based on shared metadata (*e.g.*, voucher ID). This initial dataset, containing all sequences, was analyzed using RAxML-HPC2 (Stamatakis, 2014) with ultrafast bootstrap (see below), and the resulting tree was inspected to remove co-specific terminals (*i.e.,* sister sequences with identical taxonomic identity). The pruned concatenated dataset was then used for both ML and Bayesian phylogenetic analyses. ML analyses were run using RAxML-HPC2 (Stamatakis, 2014) with ultrafast bootstrap. Bayesian inference was conducted using MrBayes v3.2.7a (Ronquist et al., 2012), applying a GTR+Γ model selected for each marker using the Bayesian Information Criterion (BIC) in jModelTest v2.1.6 (Darriba et al., 2012). MrBayes was run with 4 runs of 4 Markov chains for 30 million generations. Convergence was assessed with Tracer v1.7.2 (Rambaut et al., 2018), ensuring ESS > 200. After discarding the first five million generations as burn-in, a majority-rule consensus tree with posterior probabilities as branch support was generated using the ‘sumt’ command in the MrBayes interface. All phylogenetic analyses were performed on the CIPRES Science Gateway (http://www.phylo.org). Gene trees and the concatenated tree before pruning are all included as supplementary material (Supplemental Informations 2–6). Functions used to download and organise molecular data, as well as concatenate sequences, are available at https://github.com/amartinezgarcia/Thalassodoron.

## Results

### Systematics

**Table:** 

**Class Copepoda Milne Edwards, 1840**
**Superorder Podoplea Giesbrecht, 1882**
**Order Monstrilloida Sars, 1901**

**Thalassodoridae n. fam.** urn:lsid:zoobank.org:act:98FDDF71-7725-419B-AA59-11002CA5AAAA

**Figure 1:**
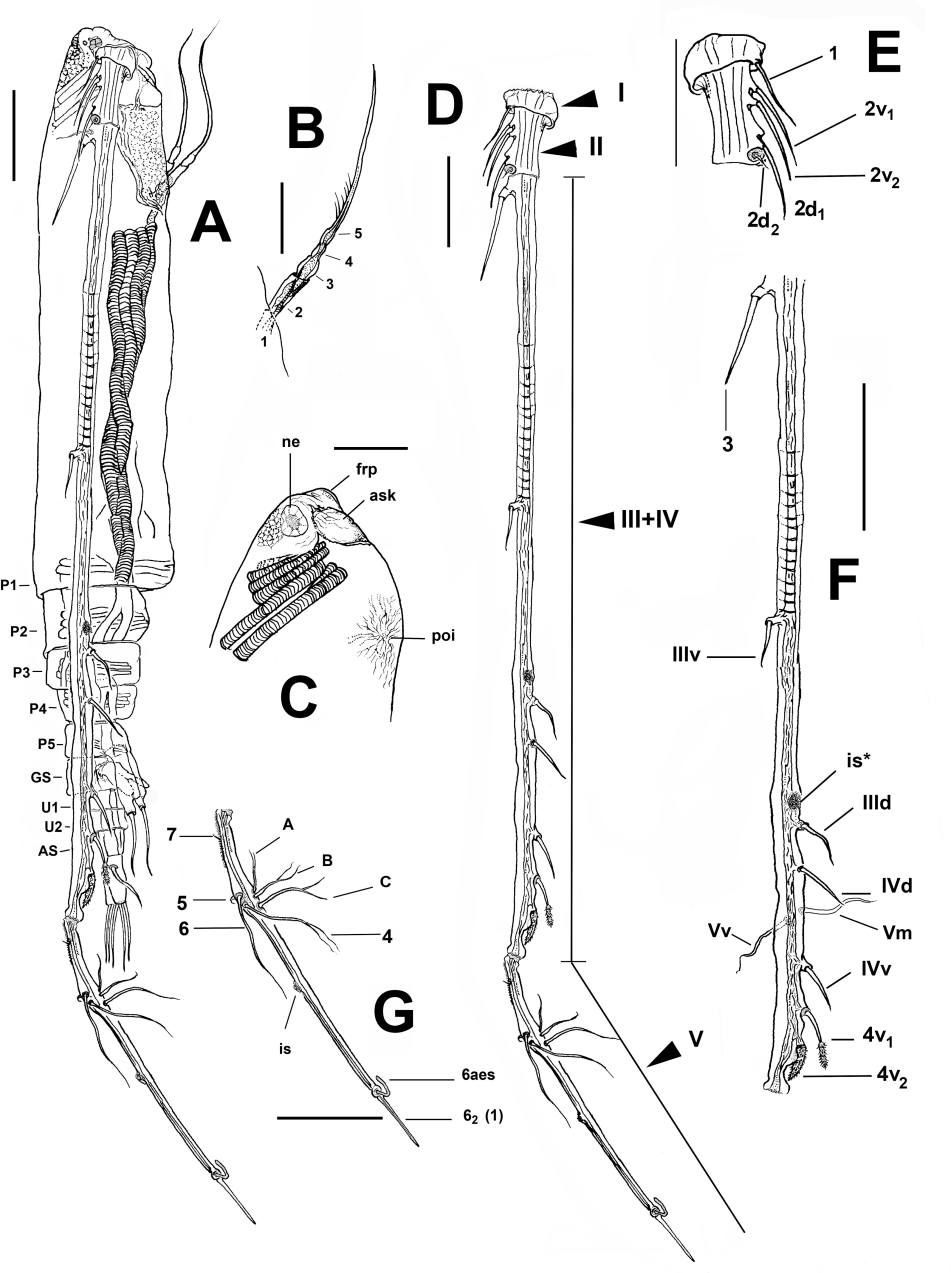
*Thalassodoron bathyale* n. gen. et n. sp. holotype. (A) Habitus, lateral view (legs 1–4 not illustrated), showing pedigerous somites PI–V, genital somite (GS), free urosomites (U1, 2), and telson (AS). (B) Perioral appendage showing segmentation. (C) Anteriormost cephalic area, lateral view, showing naupliar eye (ne), frontal protuberant process (frp), antennule insertion socket (ask), and preoral integumental process (poi). (D) Antennule showing segments I–V. (E) Antennule segments I and II showing setal armature in terms of Grygier & Ohtsuka’s (1995) nomenclature. (F) Fused antennule segments III–IV showing armature in terms of Grygier & Ohtsuka’s (1995) nomenclature. (G) Antennule segment V showing setal armature in terms of Huys et al.’s (2007) nomenclature. Scale bars: A, D–G = 500 µm, B, C = 250 µm.

**Figure 2:**
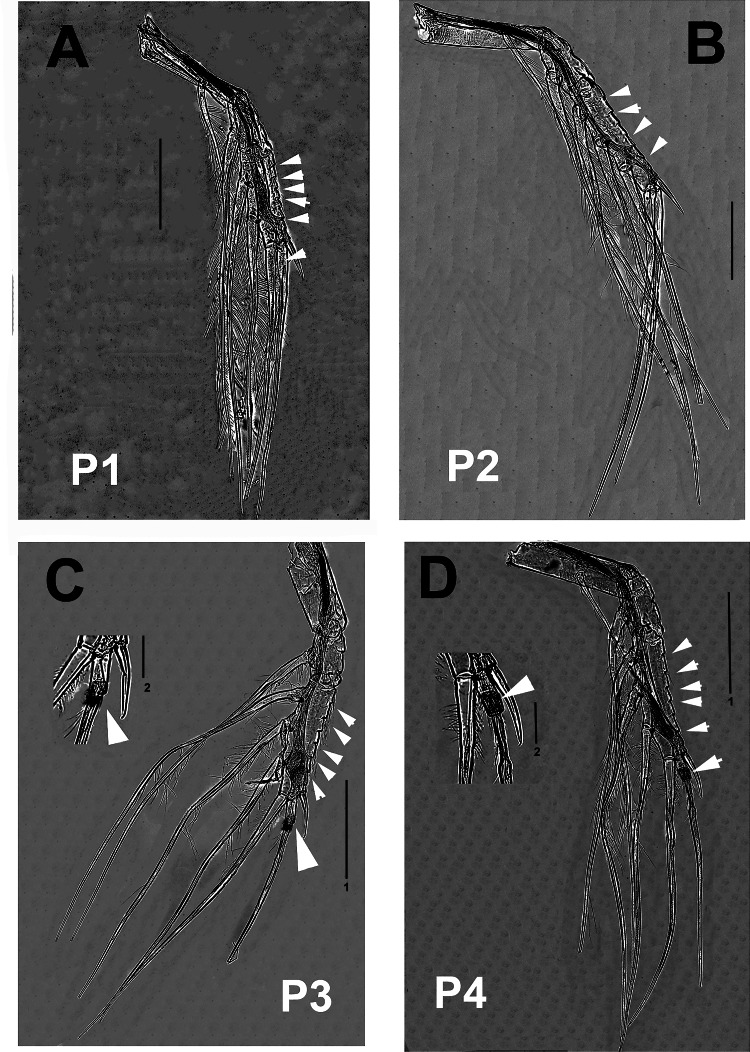
*Thalassodoron bathyale* n. gen. et n. sp. holotype. (A) Leg 1 exopod anterior view showing indentations (arrowheads) on outer margin. (B) Same, leg2. (C) Same, leg 3 with detail of proximal thickened, pilose ring on apical seta (large arrowhead). (D) Same, leg 4. Scale bars: A, B, C_1_, D_1_ = 500 µm, C_2_, D_2_ = 150 µm.

**Figure 3:**
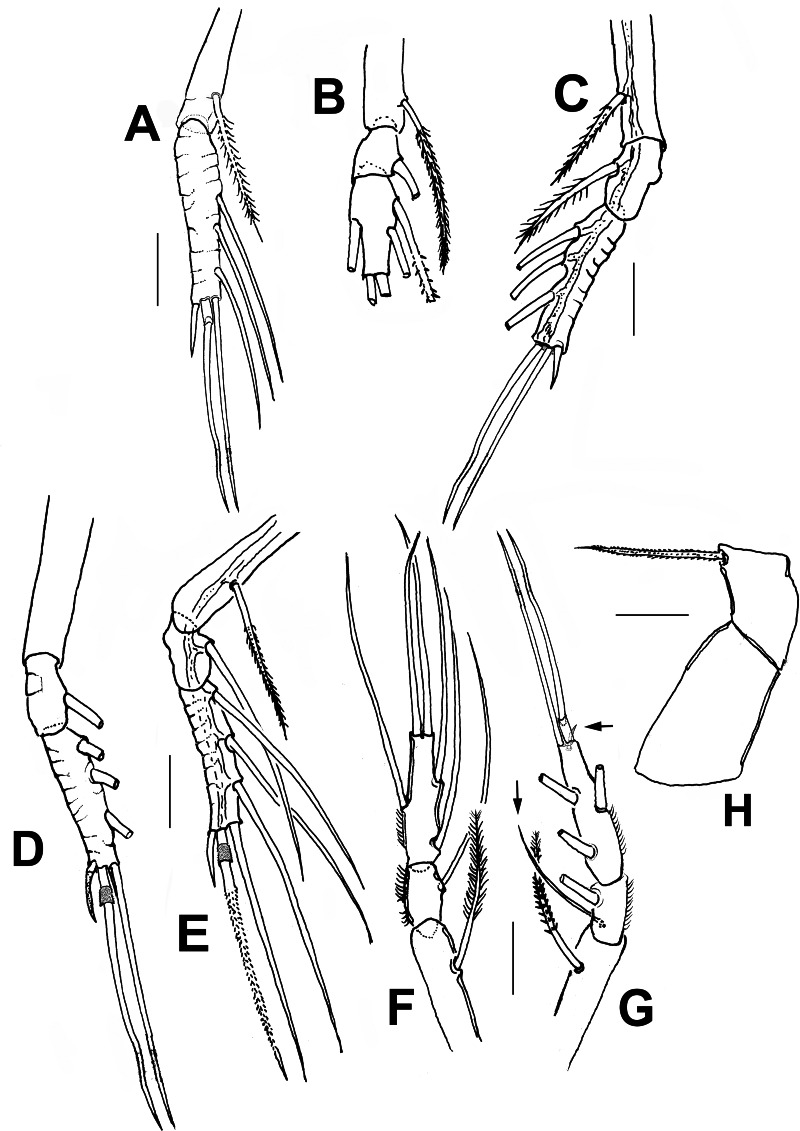
*Thalassodoron bathyale* n. gen. et n. sp. holotype. (A) Leg 1 exopod. (B) Leg 1 endopod. (C) Leg 2 exopod. (D) Leg 3 exopod. (E) Leg 4 exopod. (F) Leg 4 left endopod. (G) Leg 4 right endopod. (H) Leg 4 basipod with basipodal seta. Scale bars = 250 µm.

**Figure 4:**
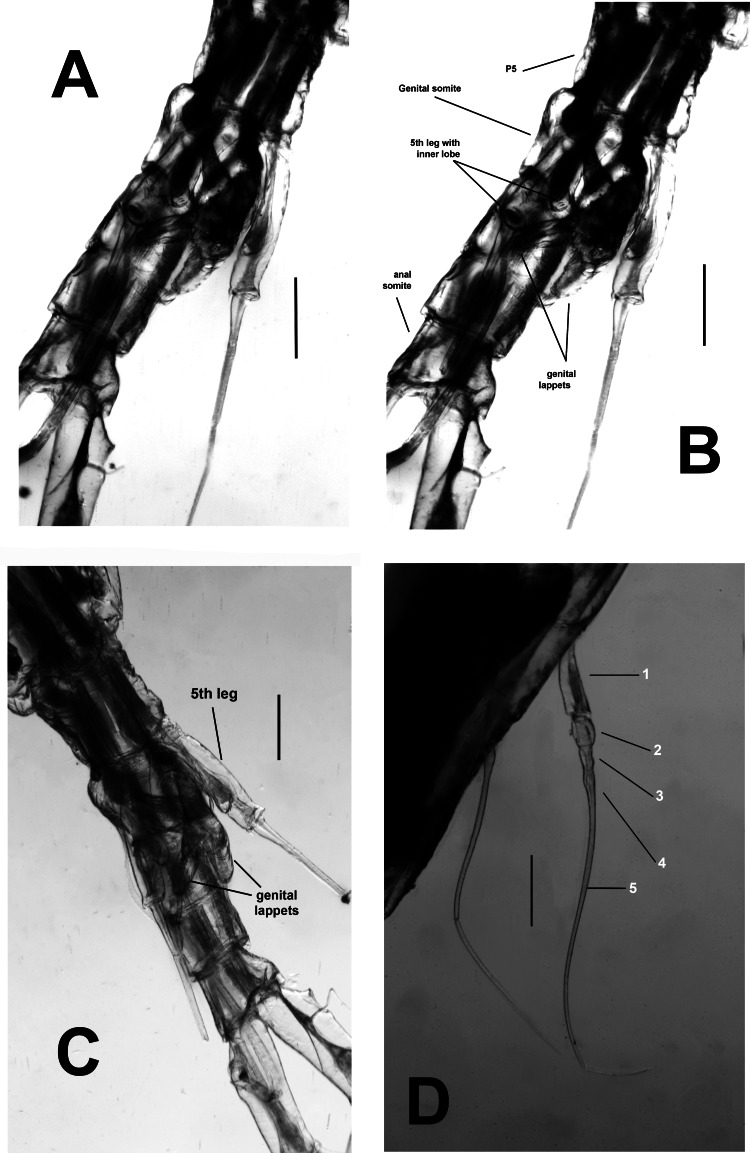
*Thalassodoron bathyale* n. gen. et n. sp. holotype. (A) Urosome, semi-ventral view showing fifth legs and genital complex. (B) Same, with labelled structures. (C) Same, ventral view. (D) Perioral appendage, lateral view, showing segmentation. Scale bars: A–C = 150 µm, D = 500 µm.

**Figure 5:**
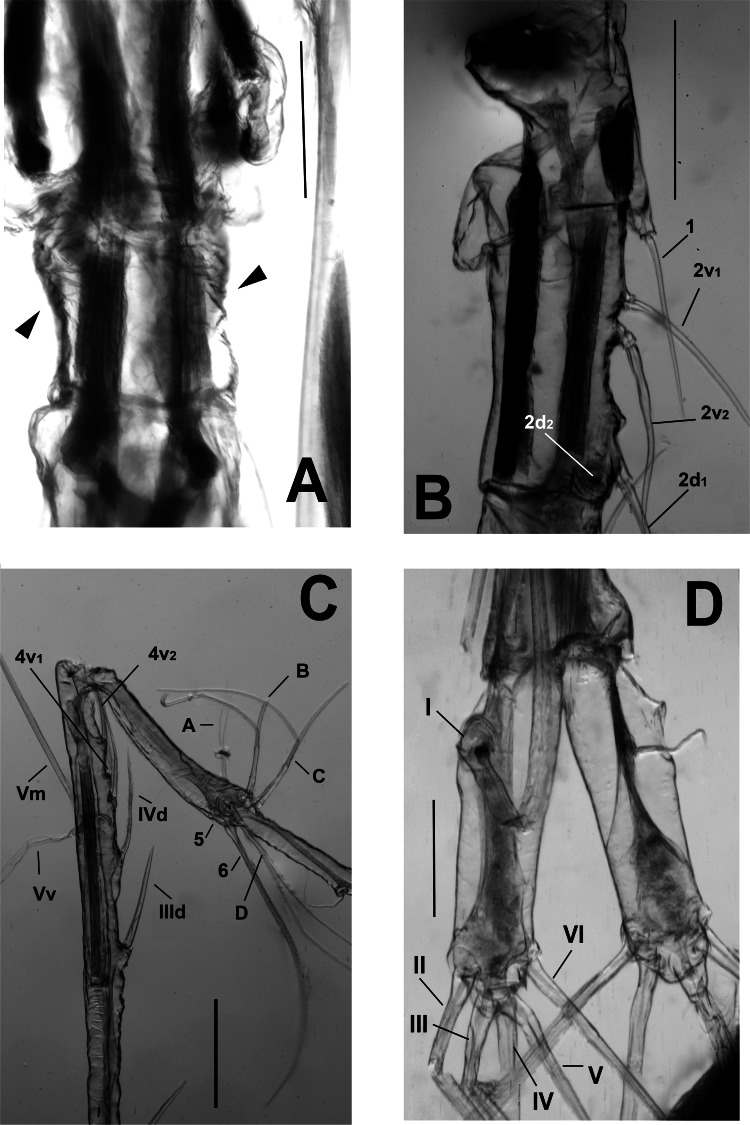
*Thalassodoron bathyale* n. gen. et n. sp. holotype. (A) Fifth pedigerous somite (P5) showing transverse integumental ridges(arrowheads). (B) Antennule segments I and II showing setal armature in terms of Grygier & Ohtsuka’s (1995) nomenclature. (C) Antennule segments III–IV (distal part) and V showing setal armature in terms of Grygier & Ohtsuka’s (1995) nomenclature. (D) Furca, dorsal view, showing caudal setae I–VI. Scale bars: A–C = 250 µm, D = 150 µm.

**Figure 6:**
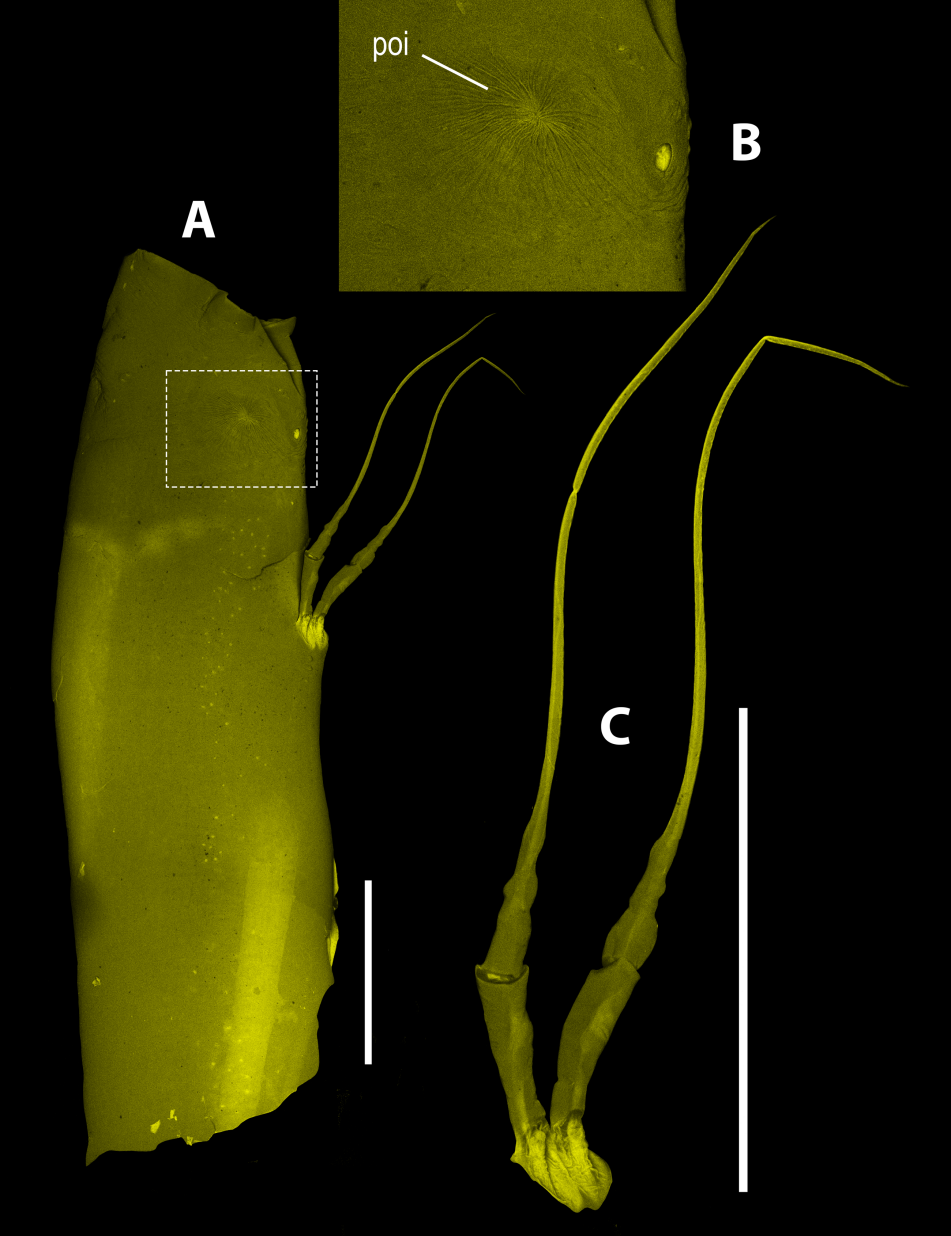
*Thalassodoron bathyale* n. gen. et n. sp. CLSM images of holotype. (A) Cephalic area, showing perioral appendage. (B) Close-up to preoral integumental process (poi). (C) Perioral appendage. Scale bars = 500 µm.

**Figure 7:**
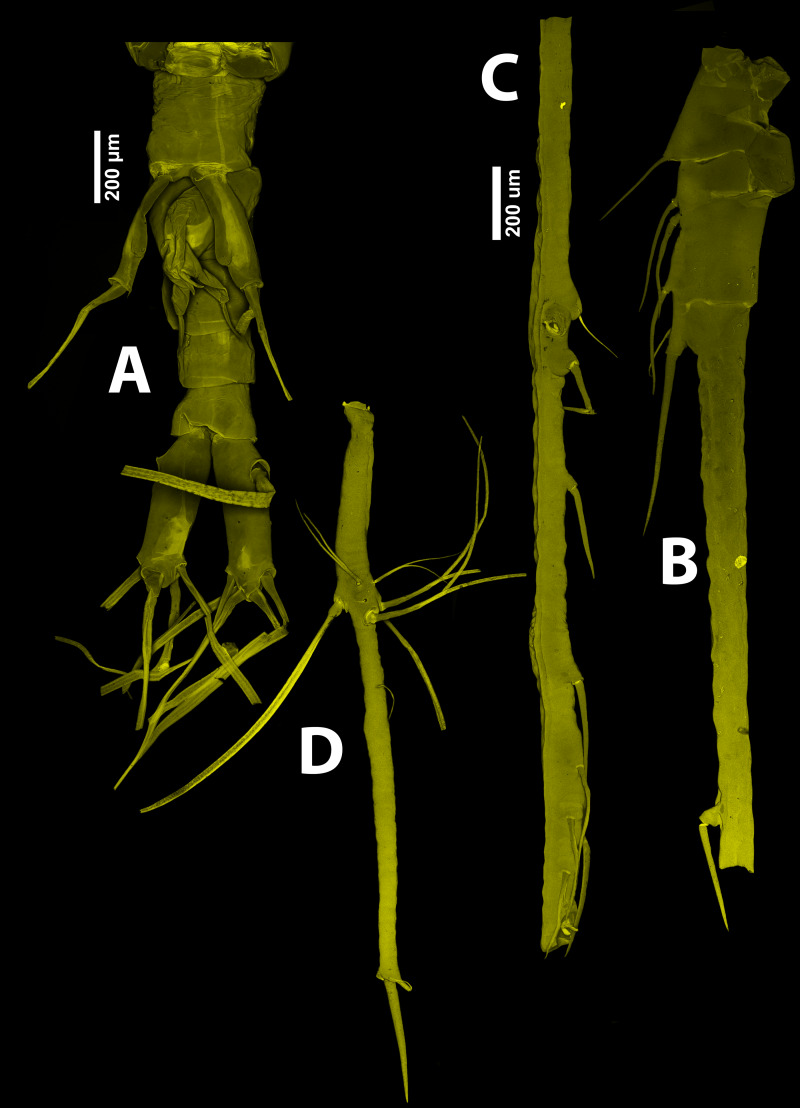
*Thalassodoron bathyale* n. gen. et n. sp. CLSM images of holotype. (A) Urosome. (B, C, D) Antennule. Scale bars = 200 µm.

The diagnosis of the new family corresponds with the diagnosis of the type genus, as follows:

**Genus** ***Thalassodoron*** **n. gen.** urn:lsid:zoobank.org:act:FA451F32-C599-4D80-885C-C2AF90ED16F4 (Figs. 1–7)

**Type species:** *Thalassodoron bathyale* **n. gen. et n. sp.**, by original designation.

**Etymology.** The name of the new genus is based on a combination of the Ancient Greek terms, *thalassa* ( 
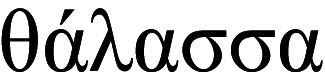
 ) = sea, and *doron* ( 
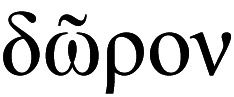
 ) = gift, meaning “a gift from the sea”, referring to the unexpected finding of this remarkable benthopelagic specimen in the North Atlantic. Epithet Latinized in nominative case, singular. Gender is neuter.

**Type material.** One adult male (ZMH-K-066757), dissected, antennule and legs mounted on permanent slides; prosome and urosome + furca preserved in 70% denatured EtOH; deposited at the Crustacea Collection of the Museum of Nature Hamburg, Zoology, Leibniz Institute for the Analysis of Biodiversity Change, Germany. Specimen partly damaged, with urosome and legs partly detached from cephalothorax; one antennule missing. The antennule broke into three parts during CLSM specimen preparation; all parts are mounted on a single permanent slide. Specimen was collected during the IceAGE expedition on board the FS Meteor, on 07 September 2011 by Saskia Brix.

**Type locality.** Irminger Basin (South Iceland), North Atlantic Ocean, Station M 85/3-1054 (61.60316N, −31.37666W; 61.61616N, −31,36,966W); Depth = 2,537.3–2,538.1 m.

**Diagnosis (based on a single male).** Large monstrilloid with long cylindrical cephalothorax, preoral ornamentation represented by field of wrinkles at insertion of antennules and pair of crater-like processes with shallow wrinkles. With low oral protuberance flanked by pair of indistinctly 5-segmented uniramous appendages. Antennule 5-segmented, geniculate, filiform, posteriorly directed, 1.3 times as long as body; segments 1–2 short. Segments 3–4 fused, remarkably long, slender. Geniculation between segments 4–5. Setal groups (*sensu* Grygier & Ohtsuka, 1995) of segments 3–4 reduced, widely separated from each other. Legs 1–4 with 3-segmented exopods and endopods. Exopods with same armature pattern in all legs; third exopodal segments of legs elongate, length/width ratio = 4.5, with regularly indented outer margin. Fifth pediger with biramous fifth leg armed with two setae on outer lobe, inner lobe thumb-like. Succeeding urosomite carrying genital apparatus with thick, slightly curved genital lappets ending in long spermatophore spines. Furca large, subrectangular, armed with six caudal setae.

**Remarks.** The new genus differs in the distinctive familial characters from all known genera of the family Monstrillidae. The antennule length, structure, and arrangement appear to be the most striking characters of the Thalassodoridae **n. fam.**; no other monstrilloid exhibits similar antennules (Figs. 1F–1G). The longest antennules known among monstrillids are found in *Monstrilla longicornis* Thompson, 1890, *M. longiremis* Giesbrecht, 1892, *M. leucopis* Sars, 1921, *M. grandis* Giesbrecht, 1891, *M. satchmoi* Suárez-Morales & Dias, 2001, and *Spinomonstrilla spinosa* (Park, 1967). In the five species of *Monstrilla* mentioned, antennules are as long as or slightly longer than the cephalothorax (Suárez-Morales, 2000a; Suárez-Morales, 2010; Suárez-Morales & Velázquez-Ornelas 2023); the antennules of *S. spinosa* are the longest among monstrillids, representing about 80% of total body length (Suárez-Morales, 2019). Antennules are, in all cases, anteriorly directed. Being 1.3 times as long as total body length, the antennule length recorded in *Thalassodoron* **n. gen.** clearly diverges from all known monstrilloids, in addition to its unique posteriorly directed arrangement, contrasting with the typical monstrillid pattern.

The new genus also differs from all other monstrilloid genera by the size and proportions of the furca. In *Thalassodoron* **n. gen.**, the furca is large, subrectangular, and remarkably long, with a length/width ratio = 3.7; also, they are 3.0 times as long as the telson. Among the monstrillid genera, species of *Cymbasoma* usually have a furca that is equally long or shorter than the telson in both males and females (Suárez-Morales & McKinnon, 2016). There are some species of *Monstrilla* with long furca, like *M. ilhoii* (Lee & Chang, 2016) and *M. grandis* (see Ramírez, 1971; Chang, 2014), nearly twice as long as the telson, but rami are quite smaller, with a length/width ratio close to 1.5, and they are even smaller in *M. grandis* males (Suárez-Morales & Üstün, 2018). The furca is almost 3.0 times as long as the telson in *M. grygieri* females (Suárez-Morales, 2000b), (length/width ratio = 1.8). Overall, the furca in the new genus is relatively longer and larger than in all other monstrilloids.

Monstrilloids are readily distinguished from the other copepod orders by their lack of oral appendages in the adult stage (Huys & Boxshall, 1991). Remarkably, *Thalassodoron* **n. gen.** exhibits a pair of indistinctly 5-segmented appendages flanking the oral area (Fig. 1B), a unique character that has not been previously reported in the Monstrilloida, in which the perioral structures are represented mainly by integumental ornamentations (Suárez-Morales & McKinnon, 2016; Suárez-Morales & McKinnon, 2025). Only a single pair of such integumental processes is found in *Thalassodoron* **n. gen.**; they likely represent attachment points of the feeding tubes active during the endoparasitic stages (Gotto, 1961; Suárez-Morales et al., 2014; Suárez-Morales, 2018). Overall, the structure (uniramous, linear segment arrangement, attenuate distal half) and perioral position of these appendages suggest that they could represent vestigial feeding appendages comparable in segmentation with a cyclopoid copepodid maxilliped (Ferrari & Ivanenko, 2001).

According to Huys & Boxshall (1991), the legs in the monstrilloid ground pattern are well developed, with 3-segmented endopodal and exopodal rami, exopods being longer than endopods. The setation pattern is highly conservative among the monstrillid genera, in which legs 1–4 are precisely alike except for one fewer seta in leg 1 exopod (Grygier & Ohtsuka, 1995). This pattern is retained in *Thalassodoron* **n. gen.** (see Figs. 2A–2D, 3A–3H). Thalassodorids lack a spine on the first exopodal segment of legs 1–4, which is present in all monstrillids. Also, in monstrillids, the third exopodal segment of legs 1–4 is subrectangular, usually with a length/width ratio ranging between 1.5 and 2.0 (Eduardo Suárez-Morales, pers. obs., 2025) and with unmodified outer margins. In *Thalassodoron* **n. gen.** the legs show some unique characters that diverge from those found in the other monstrilloid genera, *viz*.: (1) the third exopodal (legs 1–4) and endopodal (legs 2–4) segments are remarkably elongate, with a length/width ratio of 4.3–6.2, (2) the second endopodal segment of leg 4 bears two inner setae instead of one usually present in monstrillids, (3) the outer margin of the third exopodal segments is distinctly indented, thus diverging from the unmodified outer margins of the monstrillid genera; only in a few monstrillids the same margin is pilose, like in *Caromiobenella hamatapex* Grygier & Ohtsuka, 1995 and *Monstrilla pustulata* Suárez-Morales & Dias, 2001 (Suárez-Morales & Dias, 2001), and (4) the outer apical exopodal seta of legs 3 and 4 exhibits a proximal pilose ring; this character is absent in monstrillids.

The fifth leg is highly reduced or absent in males of all monstrillid genera. It is absent in *Monstrillopsis* and *Cymbasoma*. In some species of *Monstrilla* and *Caromiobenella*, the male fifth legs can be found as reduced ventral protuberances on the fifth pediger (Jeon, Lee & Soh, 2019; Cruz Lopes da Rosa et al., 2021). Only in a few species of *Monstrilla* this lobe is armed, usually with a single seta, like in the male of *M. grandis* (Suárez-Morales & Üstün, 2018), whose female shows the most ancestral armature of the fifth legs (Huys & Boxshall, 1991; Suárez-Morales, 2000a). A fifth leg bud armed with a long single seta is also found in males of *M. conjunctiva* Giesbrecht, 1902, and related species (see Suárez-Morales, 2022). The male fifth legs of the new genus are well-developed, bilobed, with a long outer (exopodal) lobe and a short, thumb-like inner (endopodal) lobe (Figs. 4A–4D). The exopodal lobe is armed with two long apical setae reaching half the length of the furca. Overall, the presence of a strongly developed, bilobed male fifth leg is an unprecedented character among monstrilloids and unique to the Thalassodoridae **n. fam.** It possibly represents an ancestral state of this appendage that was lost in monstrillids.

In *Thalassodoron* **n. gen.** the genital apparatus shows subtriangular, divergent lappets carrying long apical spermatophore spines (Figs. 4B–4C), a structure lost in male monstrillids but retained in some species of *Monstrilla*, like *M. longicornis*, *M. grandis*, *M. chetumalensis* Suárez-Morales & Castellanos-Osorio, 2019, and *M. longiremis* (Huys & Boxshall, 1991; Suárez-Morales, Carrillo & Morales-Ramírez, 2013; Suárez-Morales & Castellanos-Osorio, 2019). The medial position of the genital opercula resembles that found in both *Monstrilla* and *Caromiobenella* (Jeon, Lee & Soh, 2018; Suárez-Morales & Castellanos-Osorio, 2019).

**Table:** 

***Thalassodoron bathyale*n. gen. et n. sp.** urn:lsid:zoobank.org:act:E2D32E91-D17F-4AC0-94E2-54510AA02D91
(Figs. 1–7)

*Material examined.* Holotype adult male (ZMH-K-066757), specimen dissected, antennule and legs 1–4 mounted on permanent slides; prosome and urosome + furca preserved in 70% denatured EtOH; deposited in the Crustacea Collection of the Museum of Nature Hamburg, Zoology, Germany. Specimen damaged, with twisted cephalothorax.

*Etymology.* The species epithet comes from the ancient Greek word 
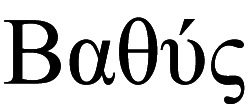
 , bathýs, meaning “deep”, and the Latin suffix -ālis (neuter -āle) to form an adjective of relationship. The name refers to the ocean depth from which this specimen was collected. The term is an adjective in the nominative singular, with gender neuter to match that of the genus.

*Description of adult male holotype*

Body size = 5.72 mm (Fig. 1A), measured from forehead anterior margin to posterior end of telson (excluding caudal setae). Body tagmosis as in male *Monstrilla* (Suárez-Morales, 2000b; Suárez-Morales, 2022; Suárez-Morales & McKinnon, 2025). First pedigerous thoracic somite incorporated into cephalothorax. Cephalothorax long, roughly cylindrical, relatively robust, widest (1.0 mm) at anterior 1/3; cephalothorax representing about 60% of total body length (Fig. 1A). Integumental wrinkles at insertion of antennules and pair of crater-like processes with slender wrinkles arranged in radial pattern. Low oral cone located at 24% of way back along ventral surface of cephalothorax, connected to long ventral digestive tube. Cephalic region anteriorly rounded, with short, thick muscle groups and rounded frontal protuberance (frp in Figs. 1C, 6A, 6B). Naupliar eye reduced, represented only by small (0.4 mm) medial unpigmented cup in the prefrontal area, lateral cups absent (ne in Fig. 1C). Cuticular ornamentation of cephalothorax including few wrinkles at insertion of antennules and pair of crater-like processes with adjacent integumental wrinkles field on ventral surface between antennule insertion and oral protuberance. Cephalothorax with low oral protuberance flanked by pair of long (1.0–1.2 mm), indistinctly 5-segmented slender appendages with attenuate distal half (Figs. 1A, 1B; 6C). First and second segments subrectangular, second segment twice as long as first; third segment with curved expanded margins, about 0.7 times as long as preceding segment. Fourth segment about half as long as third, both separated by weak constriction. Fifth segment with proximal expansion tapering distally into long, curved setiform element with row of short, slender setules (Fig. 1B).

Urosome consisting of five somites: fifth pedigerous somite (with fifth legs), genital somite (carrying genital apparatus on ventral surface), two free somites, and short telson (Figs. 1A, 7A). Fifth pedigerous somite showing transverse integumental ridges on lateral surfaces. Length ratio of urosomites (from proximal to distal) being: 22.0: 30.3: 19.1: 16.0: 12.6 = 100. Furca remarkably large, length/width ratio = 4.1, rami as long (0.47 mm) as distal three urosomites combined, armed with 6 subequally long caudal setae (I–VI), rami ornamented with few transverse integumental scars on dorsal surface. Seta IV missing in right ramus, probably lost while sampling (Fig. 4D).

Antennules inserted on prefrontal area, 5-segmented, filiform, posteriorly directed, almost 1.2 times as long as body (antennules total length = 6.67 mm), geniculate (Figs. 1A, 1D–1G, 4C; 7B–7D); first antennular segment short, subquadrate; second segment twice as long as first, segments 1–2 separated by complete intersegmental division (Fig. 1D). Segments 3–4 fused, composite segment remarkably long (2.41 mm), representing 45% of antennule length (Figs. 1D, 1F). Geniculation between segments 4–5; segment 5 elongate, slender (length = 2.1 mm) (Figs. 1G, 4C). Antennule armature including recognizable setal groups (*sensu* Grygier & Ohtsuka, 1995); setal groups widely separated from each other, as follows: first segment carrying long, setiform element 1; second segment with four elements: 2v_1_, 2v_2_, 2d_1_, 2d_2_. Combined segment 3–4 carrying elements IIId, IIIv, IVv, IVd, and pinnate, subequally long elements 4v_1_ and 4v_2_. Fifth segment long, slender; armature description follows nomenclature proposed by Huys et al. (2007) for monstrilloid males; proximal $ \frac{1}{4} $ of segment with pilose surface, with reduced setal element 7 on proximal position, setal elements 4–6 on outer margin, inner margin with branched setae A–D. Apical setal elements (*sensu* Huys et al., 2007) reduced, represented by spiniform, strongly elongate element 1 and short aesthetasc AE1 (Fig. 4C). Antennule geniculation at level of posterior end of furca. Length ratio of antennular segments (proximal to distal) as: 2.72: 5.45: 44.54: 20.92: 26.36 = 100.

First incorporated pedigerous thoracic somite and succeeding three thoracic somites each bearing well-developed biramous legs with triarticulate endopods and exopods. Legs consisting of long, wide, unarmed coxa joined by a narrow coupler with curved anterior margin; basis subrectangular, separated from coxa by complete articulation. Basis with long, stout outer basipodal seta in all legs, longest in leg 4. Coxa + basis noticeably long (1.3 mm). Legs elongate, with same armament pattern including leg 1 exopod. Basis of legs not observed. Combined length of basipod, exopod, and exopodal setae exceeding 1.5 mm in all legs. First exopodal segment of legs 1–4 elongate, cylindrical, about twice as long as succeeding second segment. Third exopodal segment of legs 1–4 elongate (0.61–0.75 mm), narrow, length/width ratio = 6.0, with regularly indented outer margins and adjacent integumental ridges, segment armed with five regular setae plus usual short outer terminal spine; outer apical seta of legs 3 and 4 with proximally thickened pilose ring. Apical setae of third exopod with spinulate outer margin, inner margin sparsely pilose. All natatory setae lightly and biserially plumose. Huys & Boxshall (1991) was followed for general morphology and setation nomenclature. Armament formula of legs1–4 as in Table 2.

**Table 2:** Armature formula of swimming legs (Roman numerals = spines, Arabic numerals = setae, * = proximally thickened seta) from outer to inner positions.

Legs	Basipod	Endopod	Exopod
Leg 1	1-0	0-1; 0-1; 1-2-2	0-1; 0-1; I-2-3
Leg 2	1-0	0-1; 0-1; 1-2-2	0-1; 0-1; I-2-3
Leg 3	1-0	0-1; 0-1; 1-2-2	0-1; 0-1; I-2*-3
Leg 4	1-0	0-1; 0-1; 1-2-2	0-1; 0-1; I-2*-3

Urosome comprising fifth pedigerous somite, genital somite, free somite, preanal somite, and telson (Fig. 7A); length ratio of urosomites (from proximal to distal) being: 26.3: 26.5: 16.4: 16.7: 14.1 = 100. Fifth pedigerous somite carrying fifth legs on ventral surface. Fifth legs strongly developed, biramous, consisting of long outer (exopodal) lobe armed with two subequally long setae reaching halfway of furca. Inner (endopodal) lobe thumb-like, reaching about $ \frac{3}{4} $ of exopod inner margin; endopodal lobe unarmed.

Succeeding genital somite with genital apparatus on ventral position, complex composed by robust, cylindrical genital shaft plus 2 subtriangular, weakly diverging lappets. Lappets with distal, slender spermatophore spine. Base of genital lappets with medial opercular flaps represented by small inner protuberances; base of shaft ornamented with pair of spiniform processes on dorsal position.

### Phylogenetic analyses

**Figure 8:**
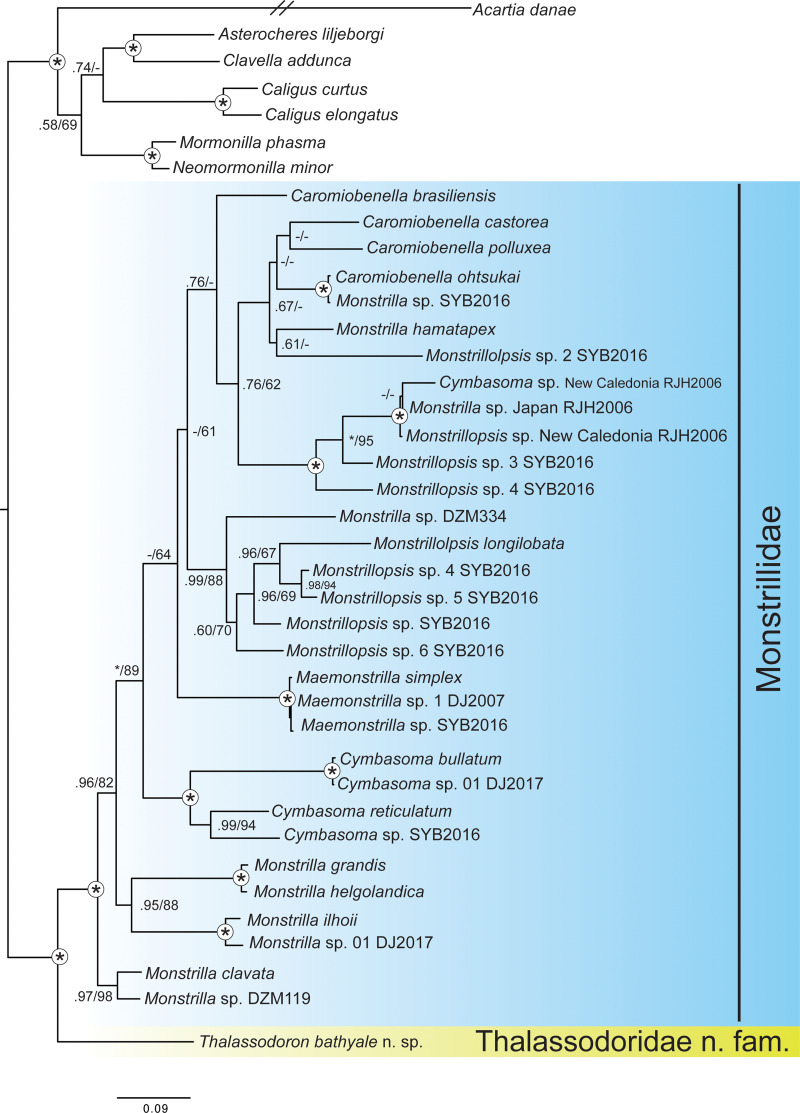
Phylogenetic relationship of *Thalassodoron bathyale* n. gen. et n. sp. based on three molecular markers (18SrRNA, 28SrRNA and mtCOI). Tree topology and branch lengths are based on the Bayesian analysis. Nodal support given above branches as Bayesian posterior probability/maximum likelihood bootstrap values. Full nodal support in both analyses is denoted with an asterisk symbol, full support in one analysis with an asterisk, and negligible support (here considered as Bayesian posterior probabilities or RAxML bootstrap lower than 0.5/50) with en-dash. Other analyses and the raw, unedited tree topologies are included as Supplemental Information.

DNA extraction and amplification yielded three sequences (mtCOI, 18S and 28S rRNA) of the holotype of *Thalassodoron bathyale* **n.** **gen. et n. sp.** The sequences of *Thalassodoron bathyale* **n.** **gen. et n. sp.** were consistently recovered as the sister group to the remaining Monstrilloida, all belonging to the family Monstrillidae (Fig. 8). This placement received maximum support both in maximum likelihood and Bayesian methods using the concatenated dataset (Supplemental Information 2–3); as well as on the 28SrRNA analyses based on Maximum likelihood (Supplemental Information 5), but not based on the 18SrRNA (Maximum likelihood bootstrap, MLB: 82) (Supplemental Information 4). Relationships within the ingroup highlight that, based on the available data and assuming the correct identification of the included sequences, most described genera within Monstrillidae might not be monophyletic.

## Discussion

The phylogenetic position of the order Monstrilloida has been difficult to establish, primarily due to the absence of mouthparts; their placement among the Copepoda, as proposed by Milne Edwards (1840), has been historically variable. Early in their study, Dana (1849) and also Claparéde (1863) included Monstrilloida as part of the tribe Monstrillacea, contained in the entomostracan suborder Cormostomata, or sucker-mouthed forms. The original description of the monstrillid genus *Cymbasoma* led Thompson (1888a) and also Scott (1889) to include it within the siphonostomatoid family Artotrogidae Brady, 1880; but, later, Thompson (1888b) proposed the family Cymbasomatidae, soon synonymized with the Corycaeidae Dana, 1846 by Bourne (1890), and this placement was also supported by Krichagin (1877) and Scott (1891). In a subsequent work, Dana (1852) moved the Monstrillidae to the tribe Ergasiloidea next to the Ergasilidae Burmeister, 1835, emphasizing the monstrillids’ obsolete mouthparts and biramous legs. Subsequently, Sars (1901) proposed the new suborder Monstrilloida to contain all *Monstrilla*-like copepods and erected a new group, the Monstrilloida Genuina, to accommodate *Monstrilla*, *Cymbasoma*, and *Monstrillopsis*. The shared lack of oral appendages and parasitic life cycle led Sars (1913) to include the semi-parasitic family Thaumatopsyllidae Sars, 1913 within the order Monstrilloida, by placing the Monstrillidae in the subgroup Monstrilloida Genuina next to the thaumatopsyllids. This classification was subsequently questioned by several authors like Davis (1949), Sewell (1949), and Fosshagen (1970) who argued that thaumatopsyllids should be placed within the Cyclopoida Burmeister, 1834, but Ferrari (1992) disagreed. Based on a set of characters of the male and female antennules and fifth legs, thaumatopsyllids were subsequently proposed by Ho et al. (2003) as the core of a new copepod order, the Thaumatopsylloida, closely linked with the Monstrilloida and Siphonostomatoida Burmeister, 1835. Boxshall & Halsey (2004) recognised the Thaumatopsyllidae only as a family of the Cyclopoida; thus, the placing of the Monstrillidae as the only family of the Monstrilloida remained unaffected. Before this debate, Gotto (1961) proposed a common ancestor of monstrilloids with the Phyllodicolidae Delamare Deboutteville & Laubier, 1961, a cyclopoid family parasitic on polychaetes, based on their type of hosts, the lack of mouthparts in the adult stage, their poorly segmented antennules, and similar feeding appendages. Huys & Boxshall (1991) considered the Siphonostomatoida and Monstrilloida as sister taxa based on their sharing of a cephalothorax incorporating the first pedigerous somite and a fifth leg lacking intercoxal sclerites. Based on 18S molecular and morphological data, and following earlier attempts to include the monstrilloids within the Siphonostomatoida (*i.e.,* Artotrogidae) (see Thompson, 1888a; Scott, 1889), Huys et al. (2007) advanced the Monstrilloida as a specialized siphonostomatoid taxon sharing a common ancestor with caligid-like taxa, both symbiotic groups diverging by their host preference, and implying the demise of the Monstrilloida as a valid order. However, the proposal of Huys et al. (2007) was not followed among the copepod community and the validity of the Monstrilloida as an order within the Copepoda remained unchanged (*e.g.* Boxshall & Halsey, 2004; Jeon, Lee & Soh, 2018; Suárez-Morales, 2022; Suárez-Morales & Mercado-Salas, 2023; Suárez-Morales & Velázquez-Ornelas, 2024).

The new family described herein, exhibits a mixture of plesiomorphic (*i.e*., vestigial condition—vestigial only for monstrilloids—of the oral appendages, presence of a strongly-developed biramous male fifth leg, geniculate, 5-segmented male antennules, complete setation pattern of leg 1 exopod, presence of spermatophore spines) and derived characters (*i.e.,* reduced number of nipple-like processes on the preoral surface, loss of basipodal seta on legs 1–4, loss of outer spine of first segment in legs 1–4 exopod, noticeably large furca). A phylogenetic analysis of these and other morphological characters, together with genetic data, should provide deeper insights into the relevance of the Thalassodoridae **n. fam.** in terms of revealing new links of the Monstrilloida with other copepod orders.

The male specimen examined here is the second largest known monstrilloid (total length = 5.72 mm), only smaller than *Cymbasoma gigas* (A. Scott, 1909) (8.2 mm total length) (see Suárez-Morales, 2001).

### Molecular position of *Thalassodoron bathyale* n. gen. et n. sp. supports its inclusion in a new family

The Bayesian phylogenetic trees calculated using maximum likelihood and Bayesian methods, and based on the mtCOI, 18S rRNA, and 28S rRNA genes, provide evidence supporting our proposal of the new family Thalassodoridae **n. fam.** to accommodate *Thalassodoron bathyale* **n. gen. et n. sp.** The family Monstrillidae was also recovered as a monophyletic group, but some monstrilloid genus groups were recovered as not monophyletic. Assuming the correct identification of the included sequences, this result was surely impacted by the missing data in many of the terminals (see Table S1), but it also highlights the importance for systematic review within the family. A robust phylogenetic hypothesis amongst the different genera within the family, out of the scope of the present study, would also require the inclusion of a morphological partition (*i.e.,* a matrix coding a separate block of morphological characters concatenated with the molecular alignments and treated as an additional partition in the analyses) to reconstruct a more robust phylogeny.

### The unique morphological characters of Thalassodoridae n. fam. associated with a benthopelagic lifestyle

The posteriorly directed antennules of *Thalassodoron bathyale* **n.** **gen. et n. sp.**, along with their considerable length and slender, seemingly fragile appearance, are unusual characters among the parasitic Monstrilloida. These features appear unsuitable to the tight confinement of the monstrilloid body within its host and the subsequent emergence through the host’s body walls. Monstrilloid copepods emerge from their hosts as preadult (Copepodid V) individuals, and thus, they experience their terminal adult molt outside the body of their polychaete host. This emergence process involves breaking the host’s body wall from within, typically by pushing out the urosome first, and ultimately releasing the antennules (see Suárez-Morales et al., 2014; Emelianenko et al., 2025). In all described monstrilloids, the stiff, anteriorly directed antennules likely facilitate this final stage of the process. However, in *Thalassodoron bathyale* **n. gen. et n. sp.**, the exceedingly long, fragile, and posteriorly directed antennules, would likely make the preadult emergence more difficult and riskier. Contrastingly, the same antennule characters, along with a large furca, may offer hydrodynamic advantages once the copepod leaves the host and is free to swim near the sea bottom. Such traits might improve swimming performance, thereby increasing the chances of locating a female in the adjacent water. Monstrilloids swim using rapid strokes of their legs (Sars, 1921; Suárez-Morales, 2018), assisted by movements of the urosome. In *Thalassodoron* **n. gen.**, the combination of long legs and a large furca likely improves swimming efficiency and appears suitable for benthopelagic life. Overall, the morphological characters defining *Thalassodoron* **n. gen.** probably enhance its reproductive success by improving swimming abilities during the non-feeding, free-swimming benthopelagic phase.

As in other zooplanktonic copepods, predator avoidance represents a high priority (Alcaraz & Strickler, 1988). It is well known that planktonic copepods can execute rapid swimming escape responses when stimulated, using synchronized thrusts of their legs, a behavior also observed in monstrilloids (see Suárez-Morales, 2018). Therefore, the long antennules and legs, as well as large furca observed in *Thalassodoron* **n. gen.**, can be interpreted as swimming traits that might also increase escape efficiency from predators in the challenging conditions of the hyperbenthos.

Copepods rely heavily on mechanoreception to detect hydrodynamic disturbances generated by approaching predators (Buskey, Lenz & Hartline, 2012). Monstrilloids usually exhibit different types of integumental sensorial structures, such as pore clusters, sensilla, or crater-like depressions (Jeon, Lee & Soh, 2018; Suárez-Morales, 2022), whose function remains unknown. In the benthopelagic environment inhabited by *Thalassodoron bathyale* **n.** **gen. et n. sp.**, one might expect a pronounced development of such sensory structures. Yet, we did not observe any special enhanced sensory features. The integumental organs located on antennule segments 3–4 appear to be the primary sensory organ in *Thalassodoron* **n. gen.**

### Lack of records for Monstrilloida in deep sea might be biased by sampling strategies

Since their initial discovery by Dana (1849), monstrilloid copepods have been recorded exclusively from a wide diversity of shallow coastal habitats, including coral reefs (Sale, McWilliams & Anderson, 1976; Grygier & Ohtsuka, 2008; Suárez-Morales, 2022) and pools in rocky shores (Cruz Lopez da Rosa et al., 2021). Recent findings of monstrilloid copepods collected at 116–700 mbsl have extended their previous known depth and habitat range (Suárez-Morales & Mercado-Salas, 2023; Suárez-Morales & Velázquez-Ornelas, 2024).

Sampling method, together with sorting taxon biases, might have an impact in the lack of reports of monstrilloid copepods in deep-sea environments. The epibenthic sledge, is known to be particularly effective for collecting benthopelagic/hyperbenthic copepods (Brandt, Van de Putte & Griffiths, 2014). Until now, monstrilloid copepods were considered exclusively planktonic organisms. However, some species, such as *Thalassodoron bathyale* **n.** **gen. et n. sp.** might show benthopelagic habits, swimming close to the sea-bottom and to their benthic polychaete hosts. Notably, the epibenthic sledge was also used to collect the two species of *Cymbasoma* described by Suárez-Morales & Mercado-Salas (2023), who also reported physical damage to their material, likely caused by the sampling process. These findings suggest that deeper waters and usually understudied habitats may harbor a greater monstrilloid diversity than previously assumed.

## Conclusions

Monstrilloid copepods are a unique and relatively understudied group of marine copepods known for their unusual life cycle and morphology. Historically, their phylogenetic relationships have been difficult to assess due to the lack of antennae and mouthparts. *Thalassodoron bathyale* **n. gen. et n. sp**. represents a remarkably distinct lineage within the Monstrilloida. The new family is distinguished from all known members of the family Monstrillidae by exhibiting a unique combination of characters including: (1) slender, posteriorly directed antennules longer than the body, (2) presence of a pair of indistinctly 5-segmented uniramous appendages flanking the oral protuberance, (3) biramous male fifth legs, and (4) large furca. Moreover, our phylogenetic analysis, including one mitochondrial (mtCOI) and two ribosomal (18S rRNA & 28S rRNA) genes, supported the assignment of a new family to accommodate the new species. The distinctive morphological characters exhibited by this new family, along with its deep-sea benthopelagic occurrence, could provide new elements for re-evaluating the phylogenetic position of the Monstrilloida, as well as insights into their biology and ecology.

## Supplemental Information

10.7717/peerj.21176/supp-1 Supplemental Information 1 List of sequences, accession numbers, and metadata used in the phylogenetic analysis

10.7717/peerj.21176/supp-2 Supplemental Information 2 Bayesian tree before pruning concatenated alignment

10.7717/peerj.21176/supp-3 Supplemental Information 3 Maximum likelihood tree concatenated alignment

10.7717/peerj.21176/supp-4 Supplemental Information 4 18S Assembly Thalassodoron-bathyale

10.7717/peerj.21176/supp-5 Supplemental Information 5 28S Assembly Thalassodoron-bathyale

10.7717/peerj.21176/supp-6 Supplemental Information 6 IQtree 28S

10.7717/peerj.21176/supp-7 Supplemental Information 7 IQTree 18S

10.7717/peerj.21176/supp-8 Supplemental Information 8 IQTree COI

## Additional Information and Declarations

### Data Availability

The following information was supplied regarding data availability: The code is available at GitHub and OSF: - https://github.com/amartinezgarcia/Thalassodoron
- Martinez, A. (2026, March 16). Thalassodoron. https://doi.org/10.17605/OSF.IO/SDGN3
The raw sequence data are available in the Supplemental Files, and at NCBI: PX412398 (18S), PX434333 (28S) The raw sequence data are available at BOLD: - ID: IACOP001-25
- BIN: AHE9355

### New Species Registration

The following information was supplied regarding the registration of a newly described species: - Publication LSID: urn:lsid:zoobank.org:pub:4092F952-8C15-454D-9924-189ADF3B2A6D - Family name: urn:lsid:zoobank.org:act:98FDDF71-7725-419B-AA59-11002CA5AAAA - Genus name: urn:lsid:zoobank.org:act:FA451F32-C599-4D80-885C-C2AF90ED16F4 - Species name: urn:lsid:zoobank.org:act:E2D32E91-D17F-4AC0-94E2-54510AA02D91
