# Supplementary figures and images for: A new family of the order Monstrilloida (Copepoda) from deep waters of the North Atlantic supported by morphological and genetic evidence
Source: PeerJ. 2026 May 18;14:e21176. doi: 10.7717/peerj.21176 (PMC13192465; doi:10.7717/peerj.21176)

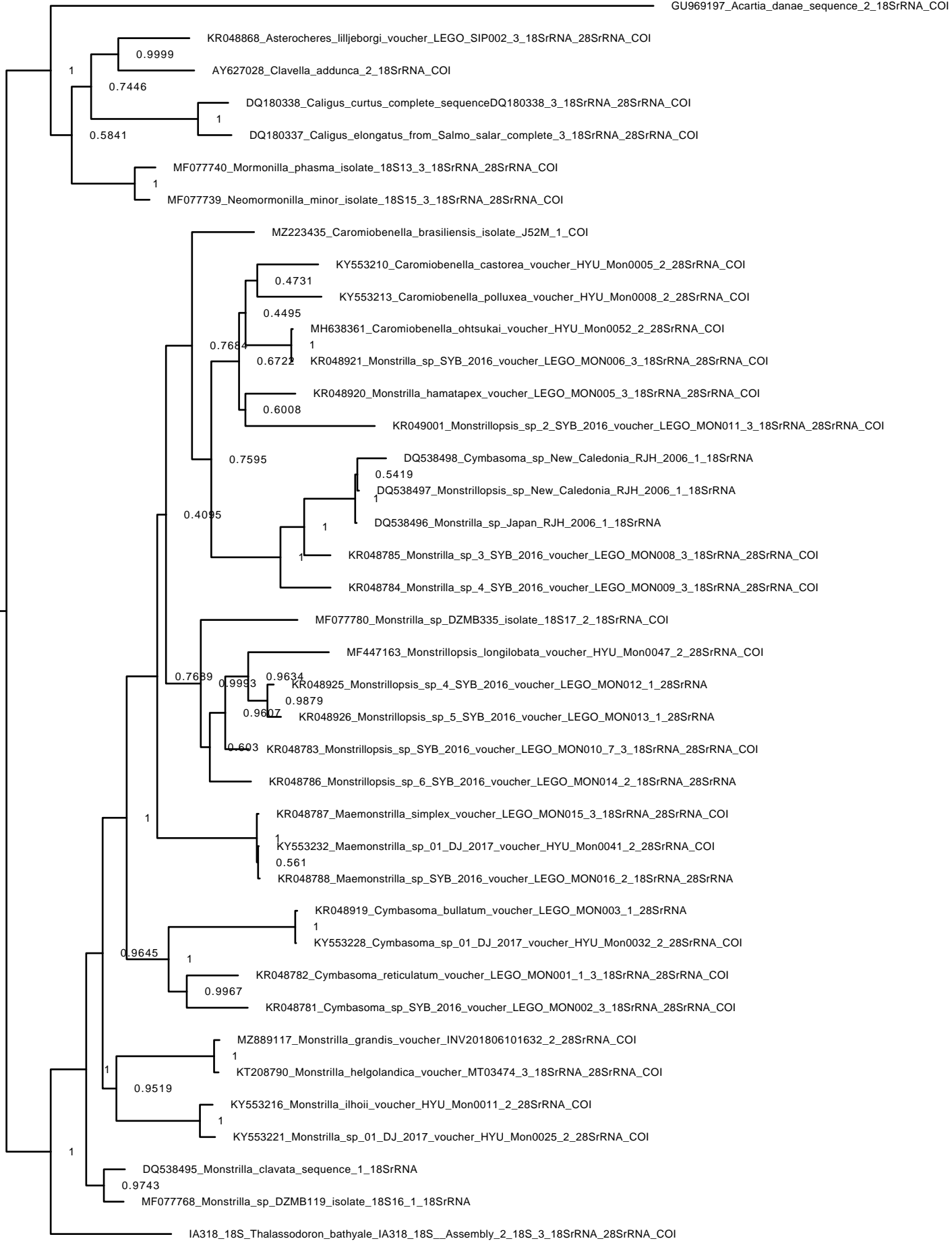

0.09

Supplement: Supplemental Information 2 [file peerj-14-21176-s002.pdf]

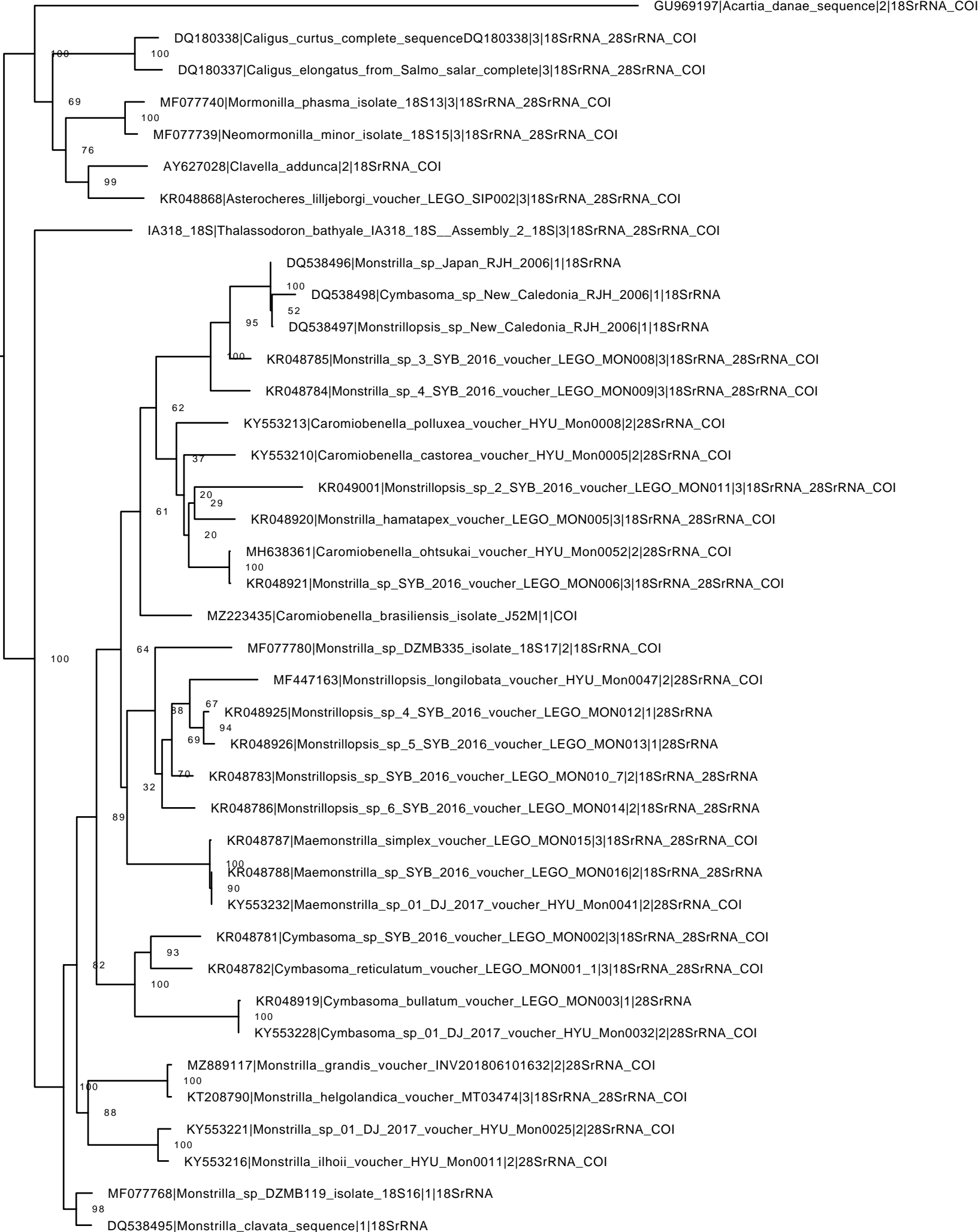

0.2

Supplement: Supplemental Information 3 [file peerj-14-21176-s003.pdf]

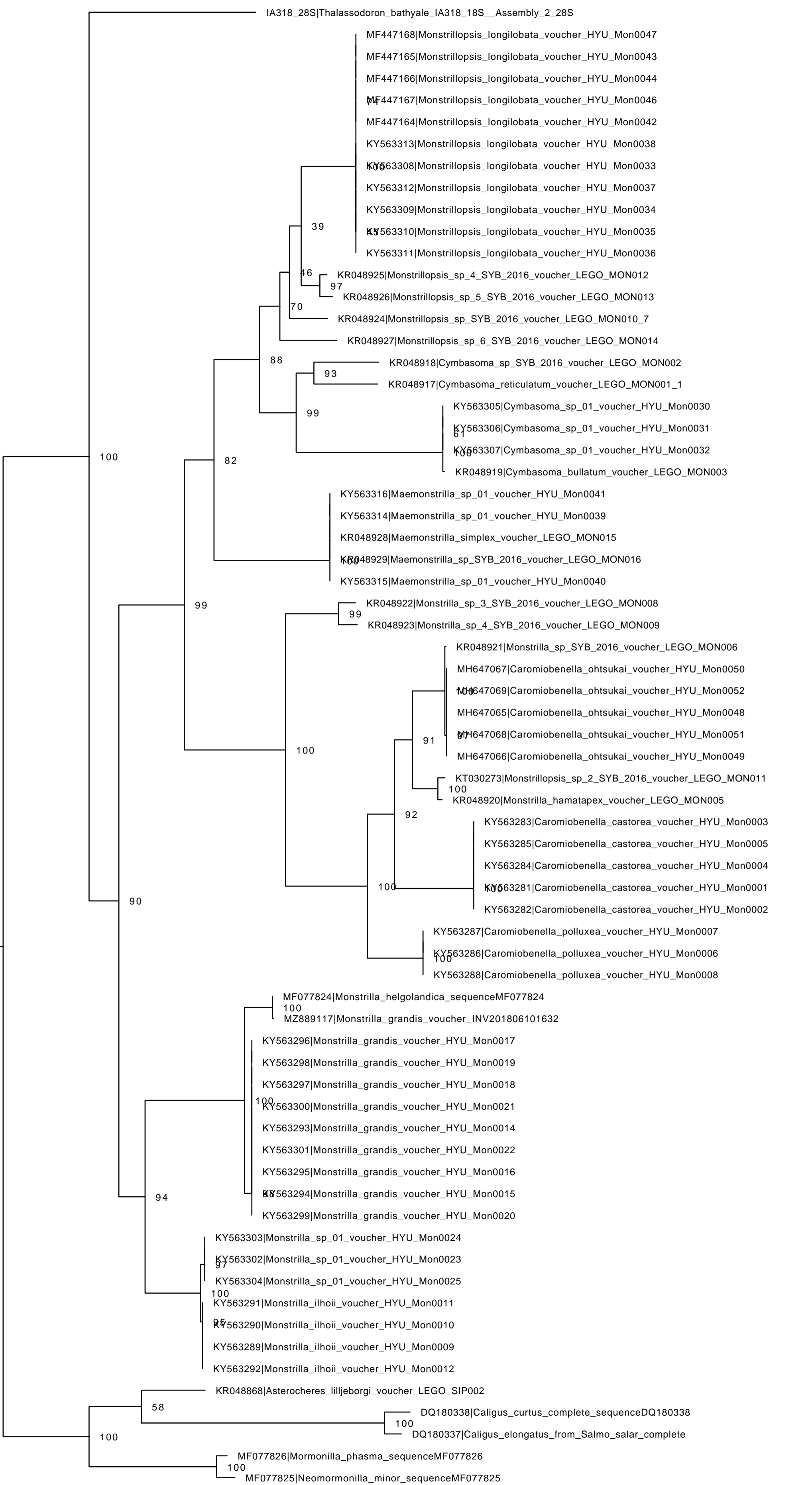

Supplement: Supplemental Information 6 [file peerj-14-21176-s006.pdf]

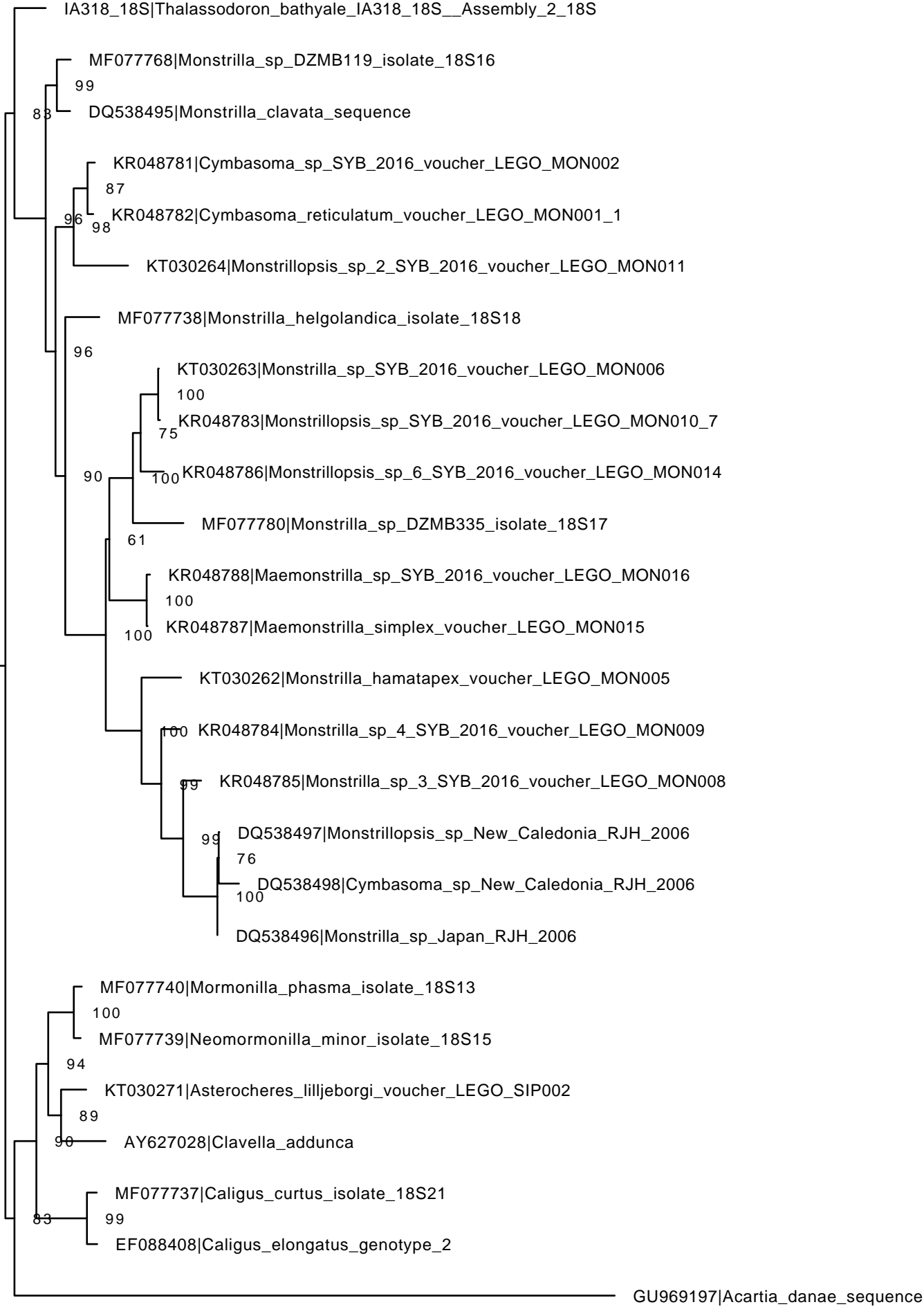

0.1

Supplement: Supplemental Information 7 [file peerj-14-21176-s007.pdf]

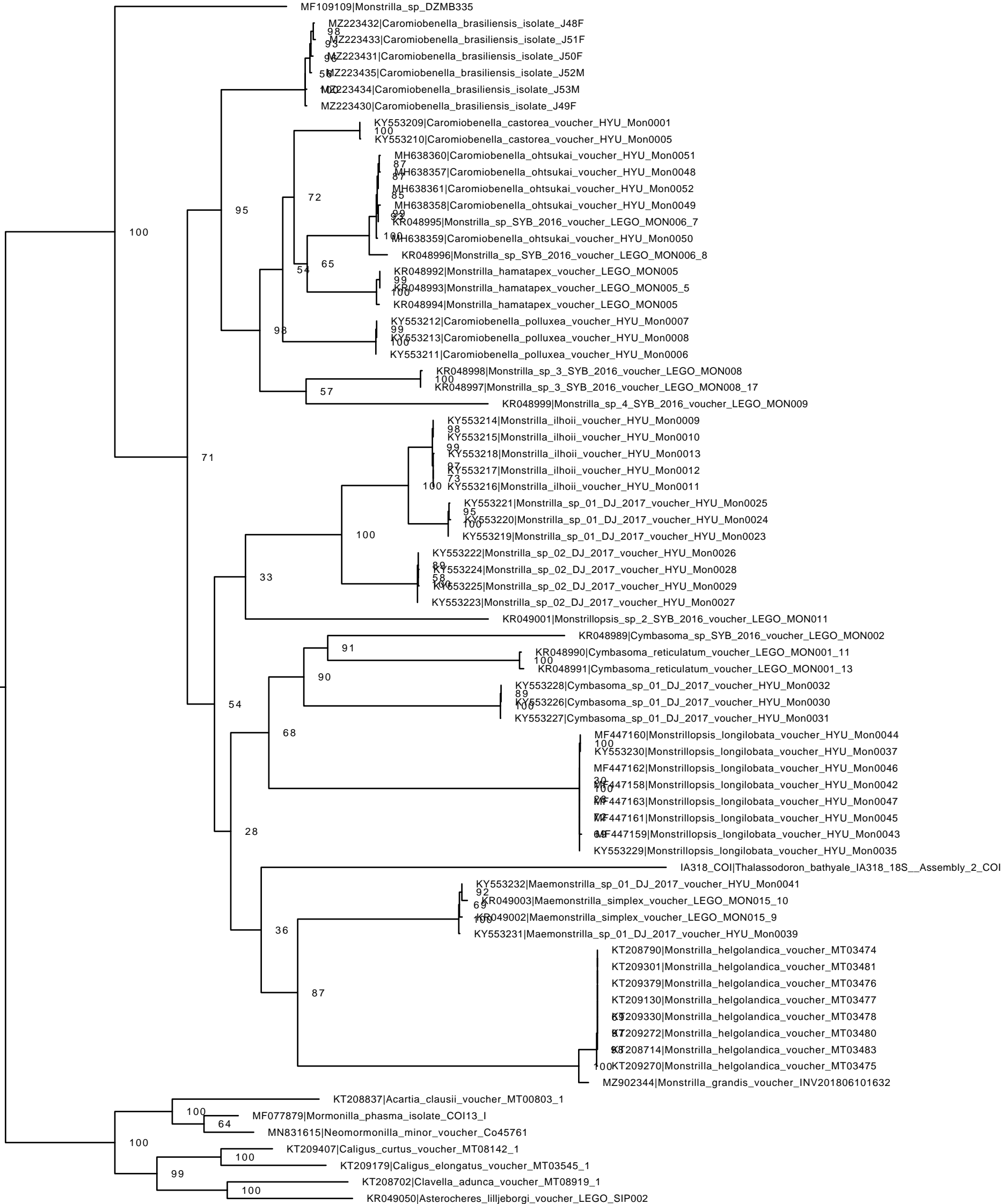

0.2

Supplement: Supplemental Information 8 [file peerj-14-21176-s008.pdf]
